# Editorial: Ethical leadership and workplace equity: mediating and moderating mechanisms in emotional labor and well-being

**DOI:** 10.3389/fpsyg.2026.1885555

**Published:** 2026-06-16

**Authors:** Friday Ogbu Edeh, Nurul Mohammad Zayed

**Affiliations:** 1 Department of Business Administration, Alex Ekwueme Federal University, Ndufu-Alike, Ebonyi State, Nigeria; 2 Faculty of Business & Communications (FBC), INTI International University, Nilai, Malaysia

**Keywords:** emotional labor, ethical leadership, leadership, well-being, workplace equity

## Introduction

Ethical leadership has gained increasing attention as a contingent condition for equity in the workplace, especially in emotionally stressful work situations where there are changing norms for employee wellbeing. Ethical leadership impacts organizational cultures that are characterized as fair, just, and respect for the dignity of the individual employee, which is grounded in fairness, integrity, and moral accountability (Edeh et al., 2025a). In modern business environments, emotional labor is necessary to attract and retain customers. Emotional labor occurs when an employee learns to control, regulate, or suppress or “act as they think they should,” their emotions to fit the role they are expected to play in their workplace or an organization (Hochschild, 1983). These emotions are acceptable thoughts in the workplace (or at least to some extent, even if they are not actually felt). For instance, a customer service employee may want to be polite to a customer even though they are frustrated within, and show some composure when speaking to an irritated customer. Hochschild (1983) classified emotional labor into surface acting: expressing and/or concealing feelings while not actually altering them. Deep acting: trying to process emotions in the way one is supposed to express them.

In this Research Topic, this trend of emerging scholarship on how ethical leadership can impact workplace equity, with complex mediating and moderating effects, is introduced, especially in the context of emotional labor and employee wellbeing. Workplace equity is the fair distribution of resources and treatment of employees. Ethical leadership promotes equity in the following ways: by demonstrating normatively appropriate behavior; by communicating clearly about decision-making; by eliminating or minimizing perceptions of bias and injustice (Hoogh and Den Hartog, 2008). The relationship between ethical leadership and wellbeing can be explained through three basic mechanisms that occur in the work environment: perceived organizational support, psychological safety, and trust (Irem et al., 2025). For example, employees' perception of ethical leader behaviors fosters positive affective states, thereby mitigating the negative impact of emotional labor. For example, some emotional labor is lessened by employees' perception of the ethical behavior of their leaders, which leads to a positive affective state.

Meanwhile, mediators such as individual differences, work characteristics, and organizational culture influence the nature and intensity of interrelationships. There is a different impact of emotional labor on wellbeing, wherein emotional labor is sometimes divided into surface acting and deep acting (Oben et al., 2025). The positive impacts of surface acting may be offset by ethical leadership, as ethical leadership can boost authenticity and lower levels of emotional dissonance, and the positive impacts of deep acting might be exacerbated by ethical leadership, as it can positively align individuals' values with those of the organization. Moreover, workplace equity may act as a direct moderator, enhancing the positive impacts of ethical leadership when fairness norms are deeply embedded.

The Research Topic “*Ethical Leadership and Workplace Equity: Mediating and Moderating Mechanisms in Emotional Labor and Well-Being*” attracted more scholars, who submitted articles aligned with its scope. Volume 1 of the above topic accepted nine (9) quality Research Papers from authors affiliated with Portugal, China, South Korea, Qatar, Malaysia, Ethiopia, South Africa, and the United Arab Emirates. The topic editors appreciate the opportunity offered by the Frontiers Team, especially during the topic formulation and review processes. The Research Topic would continue to generate positive debates amongst scholars whose interests focus on workplace equity, emotional labor, wellbeing, workplace culture, positive organizational scholarship, knowledge management practices, retention, decent work, as well as organizational learning.

This comprehensive view serves as a reminder that ethical leadership is a more complex and multifaceted process that needs to be understood as such rather than through reductionist models that emphasize a linear approach. The analysis of mediating and moderating aspects of the pathway helps closer identification of points of intervention to tackle emotional exhaustion and improve employee health and wellbeing behaviors (Edeh et al., 2025b). In this way, this strand of research has enriched the leadership theory and wider debates on sustainable and positive working environments. Creating workplace equity and ethical leadership is no longer an obligation for organizations to be concerned with; it is a business decision they make to succeed in the complex world they are operating in.

## Conclusion

Drawing from the accepted quality papers in this Research Topic, it implies that ethical leadership, when demonstrated, has the capacity to promote workplace equity and emotional labor, which engenders the wellbeing of employees. Thus, ethical leadership and workplace equity are essential in determining the dynamics of emotional labor and wellbeing. They influence interpersonal communication processes indirectly, either through influencing the perception of support and trust or through other intervening variables such as individual and organizational elements. Acknowledging these pathways can help organizations shift away from some superficial interventions and toward more comprehensive solutions that tackle the structural and psychological aspects of work. Creating ethical and equitable employment environments is ethically sound as well as necessary for improved employee resilience, a decrease in emotional load, and a sustained organization's effectiveness over time. This Research Topic is of the view that the findings drawn from the various investigations published in this volume would provide opportunities for industry policymakers and academia to benefit from the implications highlighted by the authors.
